# Enhanced photoelectrochemical aptasensing platform for *TXNDC5 gene* based on exciton energy transfer between NCQDs and TiO_2_ nanorods

**DOI:** 10.1038/srep19202

**Published:** 2016-01-18

**Authors:** Xuehui Pang, Lin Wang, Hongmin Ma, Yong Zhang, Jihong Pan, Yao Chen, Bin Du, Qin Wei

**Affiliations:** 1 Key Laboratory of Chemical Sensing & Analysis in Universities of Shandong, School of Chemistry and Chemical Engineering, University of Jinan, Jinan 250022, China; 2 Shandong Medicinal Biotechnology Centre, the Key Lab for Biotechnology Drugs of Ministry of Health, the Key Lab of Rare and Uncommon Disease, Jinan 250022, China; 3 School of Chemistry and Chemical Engineering, Shandong University, Jinan 250100, China

## Abstract

The over expression of thioredoxin domain-containing protein 5 (TXNDC5) can promote the growth of castration-resistant prostate cancer (CRPC). A novel highly sensitive photoelectrochemical (PEC) aptsensor was developed for the detection of TXNDC5 by using the nanohybrids (TiO_2_ NRs/NCQDs) of nitrogen-doped carbon quantum dots (NCQDs) and TiO_2_ nanorods as the photo-to-electron conversion medium. TiO_2_ NRs/NCQDs nanohybrids were prepared by controlling the experimental condition. TiO_2_ NRs were self-assembled to form the nanopores with good photocurrent conversion efficiency. NCQDs possessed carboxyl groups (−COOH) and amino groups (−NH_2_) in the preparation process. −COOH and −NH_2_ groups played important roles for anchoring the capture probes (5′ primer and 3′ primer) through covalent binding. The ultrasensitive and stable detection for TXNDC5 was achieved by the specific recognition between the capture probes and the targets. The fabricated aptsensor showed excellent performance with a wide linear range (0.5 fmol/L ∼ 10 nmol/L) and a low detection limit of 0.1 fmol/L. This kind of aptsensor would provide a potential application for TXNDC5.

Prostate cancer (PCa) is the second leading malignancy in men worldwide in recent years and the incidence has been increasing continuously^1^. TXNDC5 is one of the thioredoxin family involved in protein folding and chaperone activity^2^. Aberrant TXNDC5 expression has been reported in multiple malignancies. Therefore the detection of TXNDC5 is very important for the early warning of CPRC. Our group firstly systematically studied the role of TXNDC5 in CRPC^3^. TXNDC5 expressed at both mRNA and protein levels. However, no work has been done for the detection of TXNDC5. Here, we propose an aptsensor for the detection of the related expressed gene of TXNDC5based on PEC method. Various PEC aptsensors have been fabricated^4^,^5^,^6^,^7^ and PEC method is another promising DNA assay except the traditional electrochemical method^8^,^9^,^10^,^11^,^12^,^13^ due to the low background current and the high sensitivity. The separation of the excitation source and the detection signal makes this method used widely recently^14^,^15^,^16^,^17^,^18^. The aptsensor designed in this report will be helpful in the early medical clinical dignosis.

Compared with the traditional inorganic semiconductor QDs, CQDs have multiple excellent performances^19^,^20^,^21^. Most importantly, the carbon cores of CQDs are non-toxic and environmentally friendly. No abnormalities are observed in harvested organs although the amounts of CQDs found in liver and spleen are higher than those found in other organs^22^. These results clearly demonstrate that the CQDs are very desirable as the alternatives to the semiconductor quantum dots^23^,^24^. For the field of biosensing, it is very necessary to develop novel CQDs with rich surface functional groups to obtain highly effective and superior biocompatible CQD-based material. At the same time, doping is another widely used method to adjust the properties of CQDs, such as the presence of nitrogen element. N-doping effectively enhances the PEC property of CQDs^25^.

TiO_2_ is one of the most studied semiconductor nanostructures^26^,^27^,^28^. The PEC performance of TiO_2_ strongly depends on the dimensionality. Highly oriented TiO_2_ single-crystalline NRs grown on the transparent conductive substrates have been considered as the optimum choice of the solar cell materials^29^. However, a major drawback of TiO_2_ NRs is its ineffective utilization of the visible light as the irradiation source. Bandgap engineering by possible modification of TiO_2_-based materials is one of the plausible approaches to enhance the performance of TiO_2_ NRs, such as QD-sensitized.

Herein, a PEC aptsensorbased on TiO_2_ NRs/NCQDsnanohybrids as the PEC signal medium was constructed (Fig. 1). And the PEC property of TiO_2_ NRs/NCQDs was studied under the visible light. This effort offered a promising method for the detections of TXNDC5 or other analytes.

## Results and Discussion

### Characterizations of TiO_2_NRs, NCQDs, TiO_2_NRs/NCQDs nanohybirds

Scanning electron microscope (SEM)images in Fig. 2A,B showed the morphology of as-prepared TiO_2_ NRs. TiO_2_ NRs products were assembled as hierarchical microspheres as shown. Figure 2C showed a single magnified broken flower-like microsphere. It can be seen that the microsphere was composited by many uniform TiO_2_NRs with an almost average diameter about 3–5 nm. Figure 2F was EDS result. It showed only Ti, O elements in the sample, which further confirmed the prepared TiO_2_was pure. High resolution transmission electron microscope (HRTEM) was used to reveal the small size of NCQDs (Fig. 2D). HRTEM image showed that the prepared NCQDs had the plentiful output with an average diameter of 3–5 nm. And they were monodisperse nanocrystals of near spherical morphology. Figure 2E showed SEM morphology for TiO_2_/NCQDs nanohybirds. TiO_2_ NRs and as-assemble microsphere was not rigid and somewhat powder-like attachment can be distinguished on the surface. This suggested that NCQDs hybridized successfully with TiO_2_ NRs. EDS result in Fig. 2G showed four elements Ti, O, C, N, which further confirmed the excellent hybridization of TiO_2_NRs and NCQDs.

Figure 3A showed Fourier transform infrared (FT-IR) spectrum of NCQDs in the wavelength range of 500–4000 cm^−1^. The peak at 1224 cm^−1^ indicated the characteristic stretch of C-N bond. C=O bending vibration was at 1390 cm^−1^. The peak of 1625 cm^−1^ was associated with stretching vibrations of C-O band. The peak at 1730 cm^−1^ meant C=O stretching vibration. The weak peaks in the range of 2820–3050 cm^−1^ were related to C-H bond stretching vibrations. A broad peak centered at 3440 cm^−1^ suggested the presence of many hydroxyl groups on the NCQDs surfaces. All the above mentioned indicated that NCQDs had abundant carboxyl and amino groups on their surfaces. The doped amino and carboxyl groups of NCQDs can increase the hydrophilicity and the combination ability with the detection targets in aqueous systems^30^.

Figure 3B presented X-ray diffraction (XRD) patterns of NCQDs. A wide peak (002) at about 23° corresponded to an interlayer spacing *d* of 0.385 nm, which agreed with the (002) lattice spacing of carbon-based materials with turbostratic disorder^31^. *d* value was larger than that of graphite (0.34 nm), which indicated that NCQDs possessed abundant oxygen-containing functional groups^32^. But this *d* value was same to the carbon nanoparticles from pyrolyzing ethanolamine^33^ and hydrothermal carbonization of chitosan^34^.

Figure 3C displayed XRD patterns of TiO_2_ NRs, TiO_2_/NCQDs. Rutile phase TiO_2_ was produced and it can be confirmed by XRD pattern. For TiO_2_ NRs, seven diffraction peaks marked (1–7) can be respectively indexed to the (110), (101), (111), (210), (211), (220) and (002) plane. The addition of NCQDs had no obvious influence on the crystallinity and the phase purity of the resultant products. No diffraction peak resulted from NCQDs can be found, which may be attributed to the poor crystallinity and low content^35^.

### PEC performance mechanism proposition

Figure 4A showed time-based photocurrent responses of TiO_2_ NRs, TiO_2_/NCQDs under the visible light radiation. TiO_2_ could be only effectively activated under the light with short wavelength. The viewpoint was proved by the relatively weak photocurrent response even in the presence of AA in Fig. 4A (a). But the photocurrent of TiO_2_/NCQDs were apparently increased about 20 times (b), which proved the addition of NCQDs enhanced PEC behavior. This might be explained that h^+^/e^−^ pair separation efficiency was distinctly enhanced through the electron injection between NCQDs and TiO_2_ NRs.

To further explore the PEC properties of TiO_2_ NRs, NCQDs, TiO_2_/NCQDs, UV-vis absorption spectra were studied (Fig. 4B). Curve a showed a poor absorbancy of TiO_2_ NRs, which was consistent with the fact of the wide band gap of 3.2 eV^36^. NCQDs showed a broad UV-vis absorption in the ultraviolet and visible region (curve b), which suggested that NCQDs may have high photocatalytic activity. A shoulder was shown, which was at about 350 nm and was similar to another report about NCQDs 37. When TiO_2_ and NCQDs combined with each other, the absorption intensity turned stronger and the absorption range became wide (curve c), which suggested that the composition can enhance the visible light absorbancy for both TiO_2_ and NCQDs.

PL was a persuasive tool to illustrate the separation and recombination efficiency of photogenerated h^+^/e^−^ pairs. It can provide the proof for the photogenerated h^+^/e^−^ pair recombination process from another aspect^38^. Weaker PL intensity generally corresponded to stronger photocatalytic or PEC behavior. Curve a showed PL emission intensity of TiO_2_ NRs, which was obvious stronger than those of NCQDs (curve b) and TiO_2_ NRs/NCQDs (curve c). It indicated a higher recombination efficiency of the photogenerated h^+^/e^−^ pairs and a lower efficient electron injection from NCQDs to TiO_2_. PL intensity of NCQDs was lower than that of TiO_2_, which suggested that NCQDs owned better light absorbancy and photocurrent conversion efficiency. And also the addition of NCQDs into the nanohybrids decreased PL intensity which illustrated that NCQDs improved the photoelectrons transport and benefited the photoelectrons injection from occupied valence band (VB) to conduction band (CB). Therefore, NCQDs can enhance the PEC property of the wide band-gap TiO_2_ although the emission peak showed a blue shift.

The PEC performance mechanism proposition was shown in Fig. 5. A VB and an empty CB were present in the semiconductors. The electron (e^−^) was excited from VB to CB and the hole (h^+^) was formed in the VB due to the presence of a band gap under the light irradiation. However, the recombination of the electron and the hole took place generally. For TiO_2_, the photogenerated electrons more efficiently transferred to CB of TiO_2_ under the light irradiation. But subsequently the electrons were transferred directly into the VB of TiO_2_ NRs without a transition through the excited state^39^,^40^ or converted into other reactive oxidative species. And the wavelengths those were less than or equal to 380 nm can excite TiO_2_ to produce the e^−^/h^+^ pairs. However, when NCQDs were introduced into the composites system (TiO_2_/NCQDs), a mass of the visible light was converted to a shorter wavelength, which may increase the photogenerated e^−^/h^+^ pairs to enhance the PEC activity.

The relative Femi level alignment of rutile TiO_2_ and NCQDs were shown in Fig. 5. The band gap of TiO_2_ NRs was 3.2 eV^36^ and the CB energy level was −0.44 V (*vs.* normal hydrogen electrode, NHE)^41^. Then the VB energy level of TiO_2_ NRs can be deduced about 2.76 V (according to *E*_g_ = *V*_VB_–*V*_CB_). The band gap of NCQDs was about 1.9 eV^42^, the VB energy level was about 0 V^43^. So the CB energy level was about −1.9 V. Under the visible light, NCQDs absorbed the visible and near-infrared light, and then converted them to shorter wavelengths due to the up-converted property^44^,^45^. The shorter wavelengths that less than or equal to 380 nm can excite TiO_2_ NRs to produce the e^−^/h^+^ pairs. NCQDs can accept the photogenerated electrons from TiO_2_ NRs and promote the separation of the photogenerated e^−^/h^+^ pairs.

Thus, from the schematic diagram of the process, it can be seen that the VB and CB energy levels of NCQDs lied above those of TiO_2_ NRs. Under the visible light irradiation, the electrons in the VB of NCQDs obtained enough energy and injected to the CB. The photogenerated electrons can be easily injected from NCQDs into TiO_2_ NRs *via* the interface injection. However, the holes on the VB of TiO_2_ NR scan transfer to NCQDs. As a reducing agent, AA acted as an electron donor to trap the holes in the VB of TiO_2_, which inhibited the e^−^/h^+^ recombination and improved the photocurrent response^46^. After that, the electrons were transported through ITO film and conducted through the external circuit to the counter electrode.

Therefore, NCQDs in the nanocomposites facilitated the transfer of the electrons from TiO_2_ NRs and the electrons can be shuttled freely along the conducting paths in NCQDs. The combination of TiO_2_ NRs/NCQDs benefited for the charge separation and for hindering e^−^/h^+^ recombination^47^,^48^. And what’s more, N-doping was another main reason for the enhancement of the photocatalytic activity of TiO_2_/NCQDs. N-doping can lower the work function of carbon nanomaterials and the lower work function of NCQDs produced much smaller barrier between NCQDs and TiO_2_. The inference was also proved by UV-vis and PL measurement as shown in Fig. 4B,C. The above results illuminated that NCQDs can help for the transfer of the photogenerated electrons and reduce the recombination rate of e^−^/h^+^ pairs.

### Characterizations of the fabricated PEC aptsensor

In order to realize better detecting effect, the optimization of the experimental conditions was carried out. The application amount of TiO_2_ NRs, the composition methods (three methods) of TiO_2_ NRs and NCQDs, the application amount of NCQDs composited with TiO_2_ NRs, pH values and the application amount of 3′ and 5′ primers were investigated in this study (Fig. 6). The obtained optimum experimental conditions were the application amount of TiO_2_ NRs = 15 μL (Fig. 6A), the composition method was method 1 (Fig. 6B), the application amount of NCQDs = 22 mg/mL (Fig. 6C), pH = 7.4 (Fig. 6D) and the application amount of 3′ and 5′ primers = 15 μL (Fig. 6E), respectively.

The fabrication procedure of the PEC aptsensor could also be monitored by the photocurrent responses under the optimal experimental condition (Fig. 7A). PB solution contained 0.1 mol/L of AA to improve photocurrent conversion efficiency. Curve a showed there was almost non photocurrent response for bare ITO electrode. The photocurrent remarkably enhanced when TiO_2_/NCQDs nanohybrids were modified on ITO electrode (curve b). This indicated that the composition of NCQDs with TiO_2_ NRs conspicuously increased the photocurrent. Because of the composition of NCQDs and TiO_2_ NRs, the electron transfer efficiency enhanced much and the recombination probability between the electrons and the holes was depressed apparently. And also the composition manner was also investigated (Fig. 6B). TiO_2_/NCQDs prepared by method 2 showed poor PEC property with a photocurrent about 1.5 μA. TiO_2_/NCQDs prepared by method 3 displayed better PEC performance with a photocurrent about 4.6 μA. Only method 1 can make the photocurrent of TiO_2_/NCQDs improve obviously up to about 11.9 μA. The temperature and duration time using in method 1 might result in the effective combination between TiO_2_ and NCQDs. This effective combination might make the electrons injection much easier between CB and VB. Later on, when the primers of TXNDC5 were modified on the electrode, the photocurrent reduced (curve c). The bases of the primers owned−NH_2_ groups and TiO_2_/NCQDs have −COOH groups. When the primers were incubated on the modified ITO electrode surface, the interaction between the amino groups of primers and the carboxyl group of TiO_2_/NCQDs would occur under the activation of EDC/NHS amidization protocol. TiO_2_/NCQDs can immobilize the primers probes on ITO electrode through the interaction. But the bases can not transfer the electrons and absorb the visible light, which would result in the obstruction of the absorption of the light and the suppression of the electrons transfer. This would cause the decrease of the photocurrent and the experimental results proved this speculation. Therefore, the PEC aptsensor was fabricated as expected.

### Analytical performance characteristics

In order to investigate the possibility of the aptsensor applied for TXNDC5 analysis, quantitative detection of TXNDC5 was operated under the optimal conditions. The process began after incubating various concentrations of TXNDC5 on ITO electrode. The detection results were given in Fig. 7B,C. The concentration of TXNDC5 affected the strength of the photocurrent response and a stronger response was achieved at high TXNDC5 concentration. This indicated that the proposed PEC platform showed good detection performance so that it can be used for the TXNDC5 quantitative detection. A calibration graph was plotted under the optimal conditions (Fig. 7D). A positive relationship can be deduced between the photocurrent response signal change and the target concentration. PEC signal change increased linearly with the logarithm of the TXNDC5 concentration from 0.5 fmol/L to 10.0 nmol/L. The linear equation was obtained as ∆*I* (μA) = 2.901 + 0.901 lg*c*_TXNDC5_ (pmol/L) and the correlation coefficient was 0.991. A detection limit of 0.1 fmol/L was obtained for the reported aptsensor. The proposed PEC aptsensor therefore showed a wide linear range and a low detection limit for the determination of TXNDC5. The low detection limit may be ascribed to good separated excitation energy of TiO_2_/NCQDs and the specific recognition between the capture probe and the target.

Stability and reproducibility were two important parameters affecting the practical application of an aptsensor. Figure 7E showed the photocurrent responses of the PEC aptsensor with the visible-light irradiation repeated every 20 s. The irradiation process was repeated 20 on/off cycles over 800 s. During every on/off cycle, the photocurrent did not show any obvious change. This indicated that the photocurrent response was very stable and this strategy was appropriate to construct the PEC sensors. A series of six electrodes were fabricated and used to determinate 500 pmol/L of TXNDC5. All of the tests were carried out under the same conditions. The relative standard deviation (RSD) for TXNDC5 was 1.8%, so the reproducibility of the proposed PEC aptsensor was good.

## Conclusions

This work proposed a novel PEC aptsensor for the rapid and ultrasensitive detection of TXNDC5. The specific detection was realized by specific recognization between the capture probe and the target. When exposed to the visible light, the signal generator of TiO_2_ NRs/NCQDs nanohybrids expressed significantly enhanced PEC property. The combination of NCQDs improved the charge separation efficiency and the charge transfer ability, and suppressed the h^+^/e^−^ recombination effectively. The developed aptsensor displayed the ultra-sensitivity and good stability. Thus, it provided good detection effect for TXNDC5 and might provide a feasible platform to determinate of other analyses.

## Methods

### Regents

Phosphate buffered solution (PB, 0.067 mol/L KH_2_PO_4_ and 0.067 mol/L Na_2_HPO_4_) were used as the electrolyte for all electrochemistry measurements. All other chemical reagents were analytical reagent grade and directly used without further purification. The ultrapure water (resistivity of 18.25 MΩ∙cm) and pipette tips were put into a LDZX-30KBS pressure steam sterilizer (Shanghai Shenan Medical Instrument) and sterilized at 121 °C for 40 min. After cooling to room temperature, the tips were stored in a 4 °C refrigerator.

### Apparatus

SEM images were obtained from JSM-6700F microscope (JEOL, Japan). HRTEM image was recorded by JEM-2100F microscope (JEOL, Japan). FT-IR spectra were obtained with Perkin-Elmer 580B spectrophotometer (Perkin-Elmer, USA). XRD patterns were collected from D8 focus diffractometer (Bruker AXS, Germany). PEC tests were carried out on electrochemical work station (Zahner Zennium PP211, Germany). UV-vis measurements were obtained on Lambda 35 UV-vis spectrometer (Perkin-Elmer, USA). PL spectra were obtained on LS-45/55 PL spectrometer (Perkin-Elmer, USA).

### Preparation of TXNDC5

TXNDC5 was prepared according to our previous work^3^. TXNDC5 mRNA was reverse-transcribed by ReverTra Ace qPCR RT kit and SYBR Green PCR kit (Toyobo, Osaka, Japan). The primers for the amplification of TXNDC5 were as follows: forward primer 5′-CTC TGG GCC TTG AAC ATT-3′ and reverse primer 5′-CCC TCA GTG ACT CCA AA-3′. The sequence of TXNDC5 was as follows: 5′-CTC TGG GCC TTG AAC ATT CCG AAA CTG TCA AGA TTG GCA AGG TTG ATT GTA CAC AGC ACT ATG AAC TCT GCT CCG GAA ACC AGG TTC GTG GCT ATC CCA CTC TTC TCT GGT TCC GAG ATG GGA AAA AGG TGG ATC AGT ACA AGG GAA AGC GGG ATT TGG AGT CAC TGA GGG-3′. The specificity of the qRT-PCR assay was evaluated by melting curve analysis. It showed that the TXNDC5 amplification product generated a melting peak at 81.20 ± 0.34 °C without primer-dimers or nonspecific products. 15 μL of 5′ primer (10 μmol/L) and 15 μL of 3′ primer (10 μmol/L) were diluted respectively with 323 μL ultrapure water (0 °C). Then the mixtures were oscillated for 40 min under 0 °C. The concentration of the diluted primers was much greater than that of TXNDC5.

### Preparation of TiO_2_ NRs

TiO_2_ NRs were prepared according to the method in the literature with slight modifications^35^. 5 g of TiCl_3_ aqueous solution and 4 g of NaCl were added into 10 mL of distilled water under stirring. The solution was put into a Teflon-lined stainless steel autoclave with a capacity of 50 mL. The autoclave was sealed and heated at 100 °C for 12 h. After cooling to room temperature, the products were washed with distilled water and absolute ethanol for several times. Then the products dried under vacuum for use.

### Preparation of NCQDs

NCQDs were prepared as the method described in the literature with slight modifications^30^. 2 g of DTPA was placed in 20 mL ultrapure water and stirred vigorously to form a turbid liquid. Then, the formed liquid turned into the colorless solution by heating. The heating process (100 °C) was continued until the colorless solution became a yellow clustered solid, indicating the formation of NCQDs. The yellow solid was dissolved in 20 mL ultrapure water. After that, the yellow crude NCQDs solution was centrifuged at 8000 r/min for 15 min to remove the unreacted DTPA. Finally, the obtained supernatant was freeze-dried to get the pure solid of NCQDs.

### Preparation of TiO_2_NRs/NCQDs

Method 1:TiO_2_ NRs/NCQDs nanohybrids were prepared as described in the literature with slight modifications^35^. 5 g of TiCl_3_ aqueous solution, 4 g of NaCl and 10 ~ 28 mg of NCQDs powder were added into 10 mL of distilled water under stirring. The following procedure was same to the preparation of TiO_2_ NRs. TiO_2_ NRs/NCQDs nanohybrids were prepared as method 2, 3 (ESI†).

### Fabrication of the PEC aptsensor

**Figure 1:**
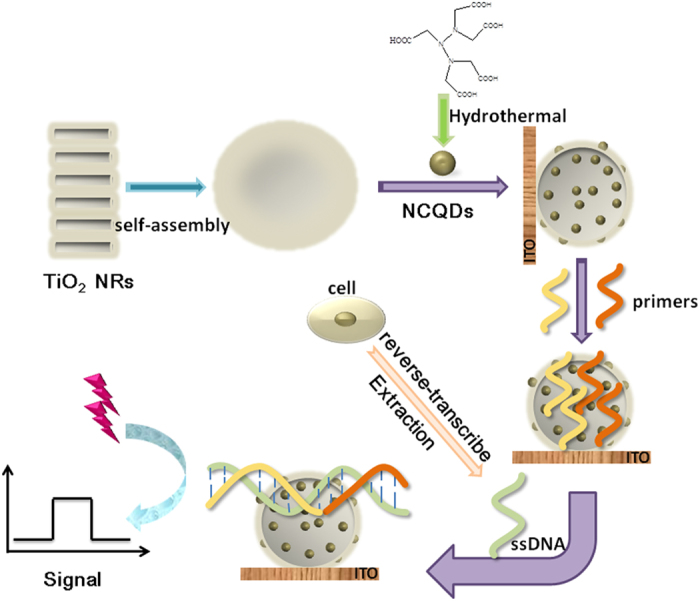
Schematic illustration of the PEC platform fabrication process.

**Figure 2:**
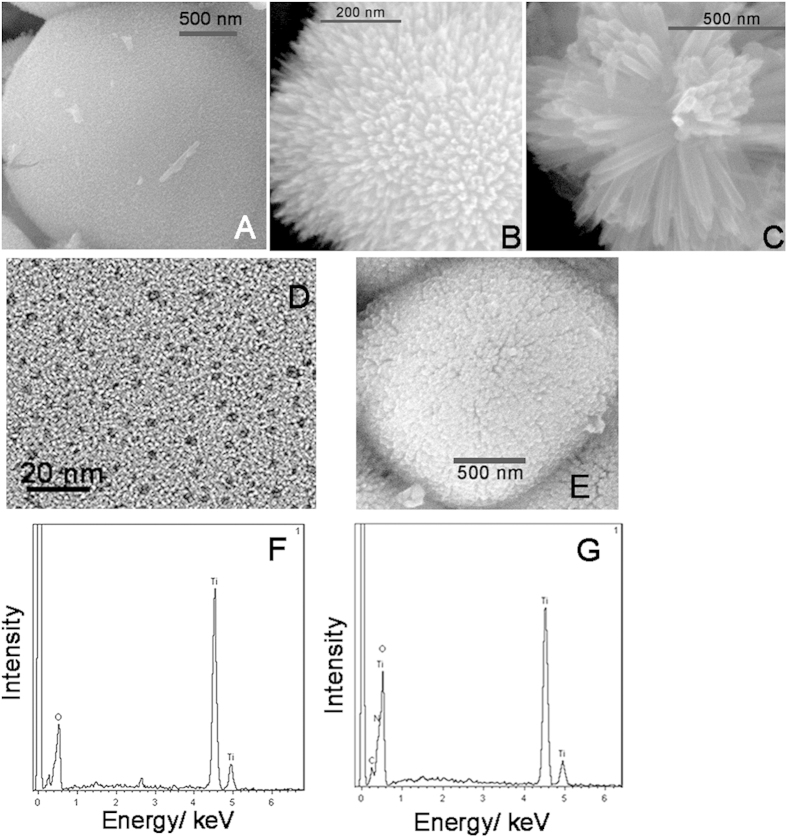
TSEM images of TiO_2_ (**A–C**) and TiO_2_/NCQDs nanohybrids (**E**).EM image of NCQDs (**D**). EDS of TiO_2_ (**F**) and TiO_2_/NCQDs nanohybrids (**G**).

**Figure 3:**
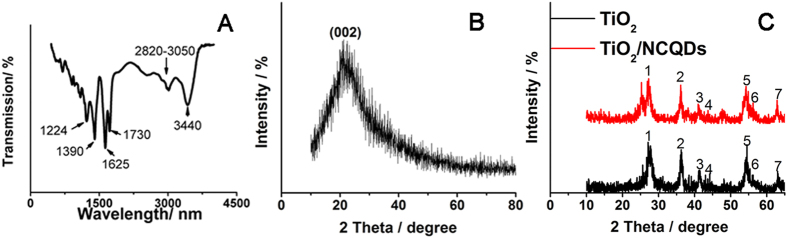
FTIR (**A**) spectrum of NCQDs. XRD spectrum of NCQDs (**B**), TiO_2_ and TiO_2_/NCQDs nanohybrids (**C**).

**Figure 4:**
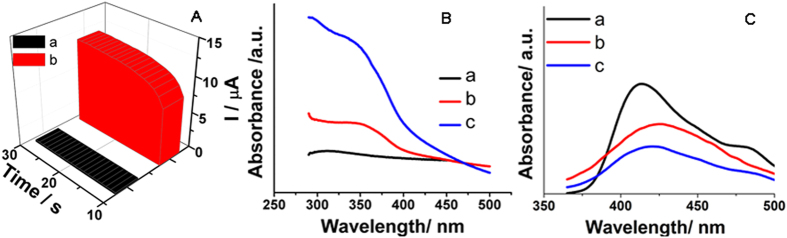
Photocurrent responses (**A**) of TiO_2_ (a) and TiO_2_/NCQDs (b) nanohybrids. UV-vis spectra (**B**) and PL emission spectra (**C**) of TiO_2_ (a), NCQDs (b), TiO_2_/NCQDs nanohybrids (c).

**Figure 5:**
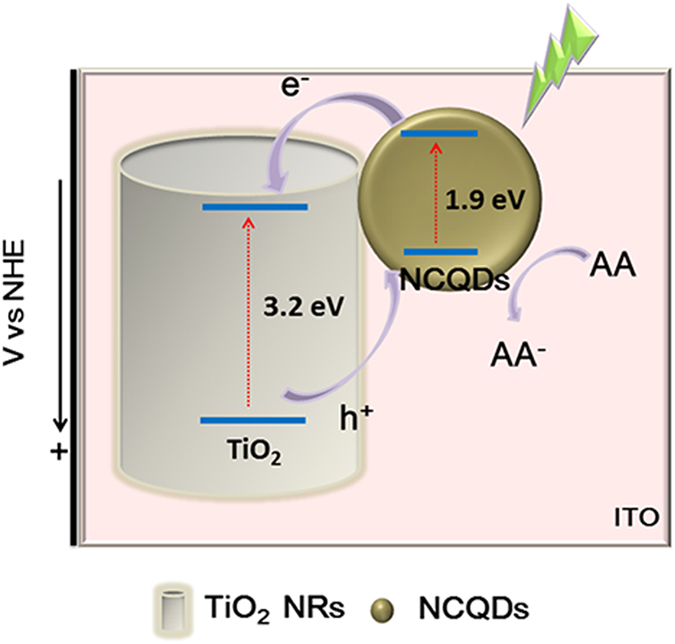
Schematic illustration of the increased photocurrent mechanism.

**Figure 6:**
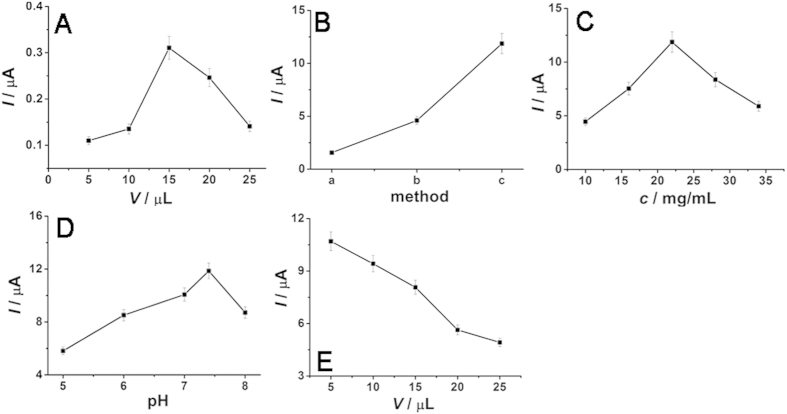
The optimization of experimental conditions by time-based photocurrent response of (**A**) different application amount of TiO_2_ NRs on ITO electrode (pH = 7.4), (B) different composition methods (a for method 1, b for method 2 and c for method 3) of TiO_2_ NRs and NCQDs, (C) different application amount of NCQDs in the composition process in the method 1, (D) different pH after modifying 10 μLof TiO_2_/NCQDs nanohybrids, (E) different application amount of 3′and 5′ primers after modifying10 μL of TiO_2_/NCQDs nanohybrids (pH = 7.4).

**Figure 7:**
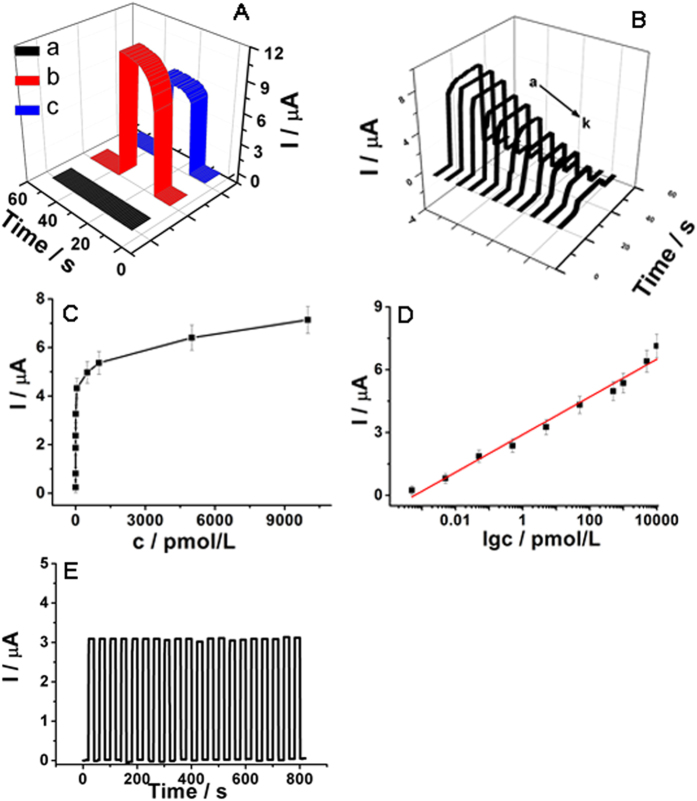
(**A**) Time-based photocurrent responses of: (a) naked ITO, (b) after modifying with TiO_2_/NCQDs nanohybrids, (c) after anchoring the primers. (**B**) Time-based photocurrent response of the aptsensor incubated with different concentration of TXNDC5; (**C**) Relation curve between photocurrent change (Δ*I*) and different TXNDC5 concentrations; (**D**) Logarithmic calibration curve between Δ*I* and *c*_TXNDC5_; (**E**) Stability of photocurrent response under the optimal conditions.

The fabrication procedure of the PEC aptsensor was shown in Fig. 1. Firstly, ITO conductive glass was cut (1.0 cm × 2.5 cm, rectangle) as the working electrode, washed orderly by ultrasonication for 30 min with acetone, ethanol and ultrapure water respectively, and dried by pure nitrogen. Secondly, 10 μL of TiO_2_/NCQDs nanohybrids were dropped on ITO electrode and dried in a 4 °C refrigerator. Thirdly, 15 μL of diluted 5′ primer was dropped on TiO_2_/NCQDs/ITO electrode surface using an EDC/NHS amidization protocol and incubated in a 4 °C refrigerator for 4 h. After that, 15 μL of 3′ primer was dropped on 5′ primer/TiO_2_/NCQDs/ITO electrode using an EDC/NHS amidization protocol and incubated in a 4 °C refrigerator for 4 h. At last, TXNDC5 ssDNA with different concentrations were dropped on 3′primer/5′primer/TiO_2_/NCQDs/ITO electrode surface respectively and incubated at 4 °C for 4 h.

### Measurement procedure

The PEC measurements were performed immediately after the incubation of TXNDC5 ssDNA. A conventional three-electrode system was used in all the PEC experiments. A platinum wire was the auxiliary electrode. A KCl-saturated calomel electrode (SCE) was the reference electrode. The modified ITO electrode was used as the working electrode. A commercial LED light (430 nm) was used as the irradiation energy in all the PEC tests. PB containing 0.1 mol/L of ascorbic acid (AA) was used as an electrolyte solution for all the PEC measurements. The bias voltage was 0.1 V. Both light duration and no light duration were 20 s. All the PEC experiments were operated at room temperature.

## Additional Information

**How to cite this article**: Pang, X. *et al.* Enhanced photoelectrochemical aptasensing platform for *TXNDC5 gene* based on exciton energy transfer between NCQDs and TiO2 nanorods. *Sci. Rep.* **6**, 19202; doi: 10.1038/srep19202 (2016).

## Supplementary Material

Supplementary Information
