# Supplementary material for: Enhanced photoelectrochemical aptasensing platform for TXNDC5 gene based on exciton energy transfer between NCQDs and TiO2 nanorods
Source: Sci Rep. 2016 Jan 18;6:19202. doi: 10.1038/srep19202 (PMC4726003; doi:10.1038/srep19202)
Supplement: Supplementary Information [file srep19202-s1.pdf]

Supplementary Material

Enhanced photoelectrochemical aptasensing platform for *TXNDC5 gene* based on exciton energy transfer between NCQDs and TiO_2 nanorods

Xuehui Pang[1], Lin Wang[2], Hongmin Ma[1], Yong Zhang[1], Jihong Pan[2], Yao Chen[3], Bin Du[1] & Qin Wei[1]*

1. Preparation of TiO_2 NRs/NCQDs

Method 2:

TiO_2 NRs/NCQDs were prepared as the method described in the literature with slight modifications [1]. 0.0040 g TiO_2 and 4 mL NCQDs solution were dissolved in 5 mL centrifuge tube and vibrated in a TCL-16 high-speed frozen centrifuge (Hunan instrument centrifuge instrument Co., LTD.) for 12 h. After that, the tube was centrifuged for 15 min under 11000 r/min and 23 ℃, then the sedimentation was collected. 1 mL ultrapure water was added in the sedimentation, The nanohybrids were ultrasonic for 20 min. Then TiO_2 NRs/NCQDs liquid nanohybrids were obtained.

Method 3:

TiO_2 NRs/NCQDs were prepared as the method described in the literature with slight modifications [1]. 0.0040g TiO_2 and 0.0040 g solid NCQDs were put in 5 mL

[1]Key Laboratory of Chemical Sensing & Analysis in Universities of Shandong, School of Chemistry and Chemical Engineering, University of Jinan, Jinan 250022, China. [2]Shandong Medicinal Biotechnology Centre, the Key Lab for Biotechnology Drugs of Ministry of Health, the Key Lab of Rare and Uncommon Disease, Jinan 250022, China. [3]School of Chemistry and Chemical Engineering, Shandong University, Jinan 250100, China. (e-mail address: sdjndxwq@163.com)

centrifuge tube, and 4 mL ultrapure water was added. The following procedure was carried out as the above method. The TiO_2 NRs/NCQDs solid nanohybrids were prepared.

Reference:

1 Pang, X. *et al.* CdSe quantum dot-functionalized TiO_2 nanohybrids as a visible light induced photoelectrochemical platform for the detection of proprotein convertase subtilisin/kexin type 6. *Biosens. Bioelectron.* **71**, 88-97 (2015).
